# Construction of Two Recombinant Pseudorabies Viruses with Deletion of Virulence Genes and Evaluation of Their Immune Protection in Mice and Piglets

**DOI:** 10.3390/vaccines13040359

**Published:** 2025-03-27

**Authors:** Shanghui Wang, Longfei Han, Jimin Yu, Guangqiang Ye, Hongyang Liu, Yunfei Liu, Qiongqiong Zhou, Zhaoxia Zhang, Changjiang Weng

**Affiliations:** 1 State Key Laboratory for Animal Disease Control and Prevention, Division of Fundamental Immunology, Harbin Veterinary Research Institute, Chinese Academy of Agricultural Sciences (CAAS), Harbin 150069, China; wangsh19961128@163.com (S.W.); 17866705591@163.com (L.H.); yujimino@foxmail.com (J.Y.); ygqyyds123@163.com (G.Y.); liuhongyang@caas.cn (H.L.); l910776357@foxmail.com (Y.L.); qqhenanly@163.com (Q.Z.); 2 College of Animal Science and Technology & College of Veterinary Medicine, Zhejiang A&F University, Hangzhou 311300, China; 3 Heilongjiang Provincial Key Laboratory of Veterinary Immunology, Harbin 150069, China

**Keywords:** pseudorabies virus (PRV), live attenuated vaccine, CRISPR-Cas9

## Abstract

**Background:** Since 2011, re-emerging pseudorabies virus (PRV) variant strains have been widespread in swine herds immunized with the classical PRV vaccine in China, suggesting that it is necessary to develop a new vaccine against these PRV variant strains. **Methods:** Here, based on a PRV mutant strain isolated in Jinmen (JM), two recombinant strains were constructed using CRISPR/Cas9 technology, including PRV-JM-ΔEK with the deletion of the *gE* and *TK* genes and PRV-JM-ΔEI92K with the deletion of the *gE*, *gI*, *US2*, *US9*, and *TK* genes. **Results:** A one-step growth curve and plaque assay revealed that the cell-to-cell transmission ability of PRV-JM-ΔEI92K was lower than that of PRV-JM-ΔEK. However, the replication ability of PRV-JM-ΔEI92K was approximately 10 times higher than that of PRV-JM-ΔEK, similar to wild-type PRV-JM. The intramuscular injection of 10^6^ TCID_50_ of PRV-JM-ΔEK or PRV-JM-ΔEI92K could not cause death in mice, and both could produce specific antibodies against gB and gD. The survival rate of mice immunized with both recombinant viruses was 100% when the mice were challenged by the PRV-JM strain. Histopathological sections from the PRV-JM-ΔEK group showed milder pathological changes compared to the PRV-JM-ΔEI92K group, proving that PRV-JM-ΔEK provided more effective protection. In pigs injected with 10^6^ TCID_50_ of PRV-JM-ΔEK or PRV-JM-ΔEI92K, their body temperature did not rise, and their weight gain was not affected. Both recombinant viruses could induce the production of gB- and gD-specific antibodies and neutralizing antibodies. After the challenge of the PRV-JM virus, neutralizing antibody production was rapidly induced and lasted for at least 3 weeks. Pigs immunized with both PRV-JM-ΔEI92K and PRV-JM-ΔEK had a 100% survival rate, demonstrating that both recombinant viruses could provide effective protection. **Conclusions:** Compared with PRV-JM-ΔEK, PRV-JM-ΔEI92K had better safety. In conclusion, we constructed two PRV recombinant viruses, which have the potential to be used as a live carrier vaccine.

## 1. Introduction

Pseudorabies virus (PRV) is the causative agent of pseudorabies, or Aujeszky’s disease (AD), which was first discovered in 1902 [1]. PRV belongs to the *Alphaherpesvirinae* subfamily of the *Orthoherpesviridae* family and is a member of the genus *Varicellovirus*. Its scientific name is swine herpesvirus type 1 (SuHV-1), and it is highly homologous to human herpesvirus type 1 (HSV-1) in evolution and belongs to the same branch [2]. The diameter of the PRV virion is about 100–150 nm, including a core, capsid, tegument, and envelope from inside to outside. The linear double-stranded DNA (dsDNA) genome is wrapped by an icosahedral capsid [3]. The PRV genome is ~150 kb in length with an average GC content of about 74%, containing at least 70 open reading frames (ORFs) that encode more than 100 viral proteins, and mature virions contain almost 50 viral structural proteins [1]. The PRV genome is large, and many genes can be knocked out and inserted into foreign genes without affecting the biological characteristics of virus replication [4].

PRV can infect pigs and a variety of other species of animals, including cats, dogs, sheep, rabbits, rats, and cattle. Pigs are the natural hosts for PRV and are the only animals that can become latent carriers. Infection with virulent PRV strains may lead to acute symptoms and even death in pigs, with the clinical signs of illness of pruritus, high fever, reproductive disorders, and encephalomyelitis [5,6]. In non-natural hosts, the mortality rate of PRV infection approaches 100% [7]. Vaccination remains the main way to control PRV-related diseases.

Live attenuated vaccines (LAVs) can stimulate pigs to produce stronger antiviral immune responses and induce higher levels of neutralizing antibodies, providing effective protection against wild-type viruses. Numerous types of commercial vaccines are currently used to control pseudorabies virus (PRV), including the *Bartha K61* strain, *Affolt 26* strain, *Ea* strain, and *SA215* strain, among others. One of the most widely used is *Bartha K61*. The *Bartha K61* strain not only lost the *gE* gene but also had many mutations in the genome of this vaccine strain: a lot of *US2* was lost, and there was a point mutation of the *gM* gene and *UL21*. The whole *gE* gene, part of the *gI* of *US9*, and the *gC* gene of the UL region exist in the *US* region [8]. There are still some hidden dangers in the *Bartha K61* strain. The main virulence gene of PRV is the *TK* gene, which is still intact, which makes the strain still have a certain virulence and can cause latent infection. Naturally, studies will show that the *Bartha K61* strain can cause swine disease [9]. PRV co-infections with other viruses, such as porcine reproductive and respiratory syndrome virus (PRRSV), classical swine fever (CSFV), or porcine circovirus type 2 (PCV2), are prevalent in most pig-raising countries worldwide [10,11]. Thus, co-infection with different viruses may result in complex interactions, potentially reducing the efficacy of the *Bartha-K61* vaccine and thus not providing complete protection against the challenge of PRV variants [12].

Until 2011, PR occurred in immune pig farms, and a mutated porcine pseudorabies virus was isolated, indicating that the existing vaccines can no longer provide complete protection against mutated PRV [13]. Since then, mutated PRV has been detected in many provinces and regions in China, such as the *ZJ01* strain, *TJ* strain, *HeN1* strain, *JS-2012* strain, *SMX* strain, *HN1201*, and so on [14,15,16,17,18]. Recently, the number of farms with pigs infected with PRV in China has increased significantly, and the epidemic of new PRV variants has posed great challenges to the prevention and control of the disease in China [19]. Moreover, several PRV infections in humans have been reported occasionally in recent years [20,21,22]. Therefore, it is urgent to develop more effective PRV vaccines that target the new PRV variants for the effective control of the disease.

A virulent PRV-JM strain was isolated. However, the serum from classic Ea-vaccinated pigs could not neutralize the virus. Interestingly, the serum from pigs infected with the PRV-JM strain could neutralize other PRV strains, including the Tianjin (TJ) strain [23]. In this study, two recombinant viruses, PRV-JM-ΔEK and PRV-JM-ΔEI92K, were constructed and derived from the PRV-JM strain by the CRISPR/Cas9 system. Subsequently, their safety and immune efficacy were evaluated in both mice and pigs. Based on its safety, protection, and strong replication ability, PRV-JM-ΔEI92K was ultimately chosen as a candidate vaccine for controlling PRV. It is possible to use PRV-JM-Δ EI92K as a living vector to express the main immunogenic proteins of other pathogens.

## 2. Materials and Methods

### 2.1. Viruses, Cells, and Plasmids

PK-15 (bio-73128), HEK293T (bio-72947), and Vero-E6 (Bio-108895) cell lines were obtained from ATCC. They were cultured in DMEM (Gibco, Grand Island, NY, USA) supplemented with 10% fetal bovine serum (FBS; Gibco, Grand Island, NY, USA) in 5% CO_2_ at 37 °C. The PRV-JM strain (OK338077.1) was isolated from an aborted pig sample of a PRV-positive pig farm in Jinmen, Guangdong Province, in 2021 and was amplified in PK-15 cells [23].

### 2.2. Primers and Plasmid Construction

sgRNAs were designed according to the online CRISPR Design Tool (http://crispor.tefor.net/, accessed on July 31, 2023).and targeted the PRV *gE*, *gI*, *US2*, and *TK* gene open reading frames and EGFP expression cassettes (Table S1). The pX330 plasmid digested by the *Bbs* I enzyme (New EnglandUS2 Biolabs, Beijing, China) was used to construct a homologous recombinant intermediate transfer vector. The PRV-JM strain *gE*, *TK* gene open reading flanking homology arms, and the EGFP expression cassette were amplified by PCR (Table S2). A multi-fragment recombination method was used to construct pUC19-*gE*-EGFP and pUC19-*gE*. Identification primers were designed on both sides of the *gE*, *TK*, *US2*, and *gI* genes (Table S3). The PCR primer synthesis in this study was performed by Comate Biotechnology Co., Ltd. (Jilin, China).

### 2.3. The Extraction of the Complete Full-Length Genome of the Virus

The PRV-JM strain was inoculated into monolayers of Vero-E6 cells and cultured at 37 °C. The cells were centrifuged at 4 °C at 250× *g* for 10 min when the cytopathic effect (CPE) involved at least 80% of Vero-E6. Then, the full-length genome was extracted from cultured cells, as previously described [24], and stored at −80 °C.

### 2.4. Construction of Viruses

Recombinant viruses were constructed by a genome co-transfection method. Vero cells were plated into a 6-well cell culture plate. When the cells were 80% confluent, the cells were co-transfected with 2 µg of viral genomic DNA, 1 µg of plasmid pX330-sgRNA, and 1 µg of the pUC19 intermediate transfer vector, together with a lipo3000 transfection reagent. After 3–4 days of transfection, when the cells showed about 80% lesions, the 6-well cell culture plate was repeatedly frozen and thawed three times, and the virus was collected. Viruses diluted by 10^2^, 10^3^, and 10^4^ were inoculated into a 6-well cell culture plate filled with monolayer PK-15 cells and incubated at 37 °C and 5% CO_2_ for 2 h. The virus was purified via plaque purification in PK-15 cells overlaid with MEM containing 1% low-melting-point agarose and 2% FBS. After 36 h, when obvious spots appeared, purified clones were randomly picked and further propagated. Positive virus clones were identified by PCR. The constructed gene deletion strains were named PRV-JM-ΔEK and PRV-JM-ΔEI92K.

### 2.5. In Vitro Growth Properties and Plaque Morphology

To measure the growth curve of the virus, PK-15 cells cultured in 6-well plates were inoculated with PRV-JM-ΔEK, PRV-JM-ΔEI92K, and the parental virus JM at a magnification of infection (MOI) of 0.1. After incubation at 37 °C for 2 h, the supernatant was discarded and washed once with PBS, and DMEM with 2% FBS was added. At 6, 12, 24, 36, 48, and 60 h after infection, the supernatant was harvested, and the virus titer was determined by a 50% tissue culture infection dose (TCID_50_). Plaque sizes were determined at 72 hpi in PK-15 cell monolayers. After infection by JM, PRV-JM-ΔEK, and PRV-JM-ΔEI92K for 2 h, the cells were overlaid with 1% low-melting-point agarose in MEM supplied with 2% FBS for plaque formation. Then, the cells were stained with 1% crystal violet for 12 h and observed at room temperature.

### 2.6. Safety and Protection Assessment in Mice

To verify the safety of the deleted strains, 4-week-old female BALB/c mice were randomly divided into 13 groups, with 5 mice in each group. The first 12 groups were injected with viral suspensions (10^3^ TCID_50_, 10^4^ TCID_50_, 10^5^ TCID_50_, and 10^6^ TCID_50_) of JM, PRV-JM-ΔEK, or PRV-JM-ΔEI92K via intramuscular injection. The 13th group of mice was injected with DMEM as a control group. The mice were observed continuously for 28 d, their survival status and clinical signs of illness were recorded, and blood samples were collected every week. The surviving mice were sacrificed on day 28 post-injection to determine the pathological change in the spleen, lungs, kidneys, and brain.

Female BALB/c mice at 4 weeks of age were randomly divided into 3 groups, 5 mice in each group. The experimental groups received intramuscular injection with 10^6^ TCID_50_ of PRV-JM-ΔEK or PRV-JM-ΔEI92K. The control group was injected with 100 μL DMEM. After 28 d of immunization, the mice were challenged with 10^4^ TCID_50_ of the JM strain. The clinical signs of illness and deaths of the mice were recorded every day. Blood samples were collected every week. At 14 d post-challenge, the surviving mice were sacrificed, and their spleens, lungs, kidneys, and brains were collected to determine the pathological changes.

### 2.7. Pathogenicity and Immunological Experiments in Pigs

One-month-old pigs free of PRV, porcine reproductive and respiratory syndrome viruses (PRRSVs), and porcine circovirus 2 (PCV2) were randomly divided into 3 groups. A PRV gB competitive ELISA kit, PRRSV indirect ELISA kit, and PCV2 indirect ELISA kit were all purchased from jinnuo diagnosis (Beijing, China). Blood was collected from the anterior vena cava of each pig, and after standing at 37 °C for 30 min, it was centrifuged at 500× *g* for 5 min to collect serum. The serum samples were tested according to the kit instructions, and three replicates were used for each pig to ensure accurate results. The pigs were intramuscularly administered with 10^6^ TCID_50_ of the PRV-JM-ΔEK or PRV-JM-ΔEI92K virus and inoculated with DMEM, serving as negative control. All pigs were challenged with 2 × 10^5^ TCID_50_ of the JM strain through intranasal instillation at 28 dpi. Furthermore, the clinical signs of illness, rectal temperature, and the death of all pigs were recorded daily. Body weight was recorded every week. The pigs were dissected immediately after death; their liver, spleen, lungs, brain, and lymph nodes were collected; and lesions were observed and photographed. At 21 d post-challenge, all pigs were euthanized and necropsied, and organ samples were collected. The lesions were observed and photographed.

### 2.8. ELISA Detection of gB- and gD-Specific Antibodies

The gB and gD proteins of PRV (expressed by our laboratory through an insect cell expression system) were diluted to 0.2 μg/mL in coating buffer and coated on high-binding 96-well ELISA plates. The plates were then incubated at 37 °C for 5 h and blocked with 5% skim milk for 2 h. The serum samples (1:200 dilution) were added to 100 μL/well. Positive and negative serum samples were included in each run. The plates were incubated at 37 °C for 30 min and washed three times, and HRP-labeled anti-mouse or -pig (1:8000 dilution, 100 μL/well) was added to all the wells of the plates and incubated at 37 °C for 30 min. TMB substrate solution was added to each well (100 μL/well) and incubated at 37 °C for 15 min. The reaction was stopped by adding 0.5M HCl. Then, the OD_450nm_ value was measured.

### 2.9. Neutralization Tests

The serum samples were inactivated at 56 °C for 30 min and then were diluted in a 2-fold serial dilution with DMEM. Each was mixed with 200 TCID_50_ PRV solution and incubated at 37 °C for 1 h. Subsequently, the mixture was added to 96-well plates lined with Vero cells and incubated at 37 °C for 2 h. The supernatants were changed to DMEM containing 2% FBS and cultured at 37 °C for 2–5 days. The cytopathic effect (CPE) was observed under a microscope. Finally, serum neutralization potency was calculated using the Reed–Muench method.

### 2.10. Histopathological Examination

Histopathological examination was performed as previously described [25]. The samples were fixed with aqueous formalin solution for 24 h, after which they were embedded in paraffin wax, and sections of 3 µm size were cut and stained with hematoxylin and eosin (H&E). Finally, the samples were observed with a light microscope.

### 2.11. Statistical Analysis

Data were processed and analyzed by GraphPad Prism software (GraphPad Prism Version 5, GraphPad Software, La Jolla, CA, USA, 2012). The normality of the data was tested using the Shapiro–Wilk normality test. Statistical analyses between different groups were performed using a one-way Analysis of Variance (SPSS software, version 25.0.0.0). *p*-values < 0.05 were considered statistically significant.

## 3. Results

### 3.1. Construction of Recombinant Viruses

**Figure 1:**
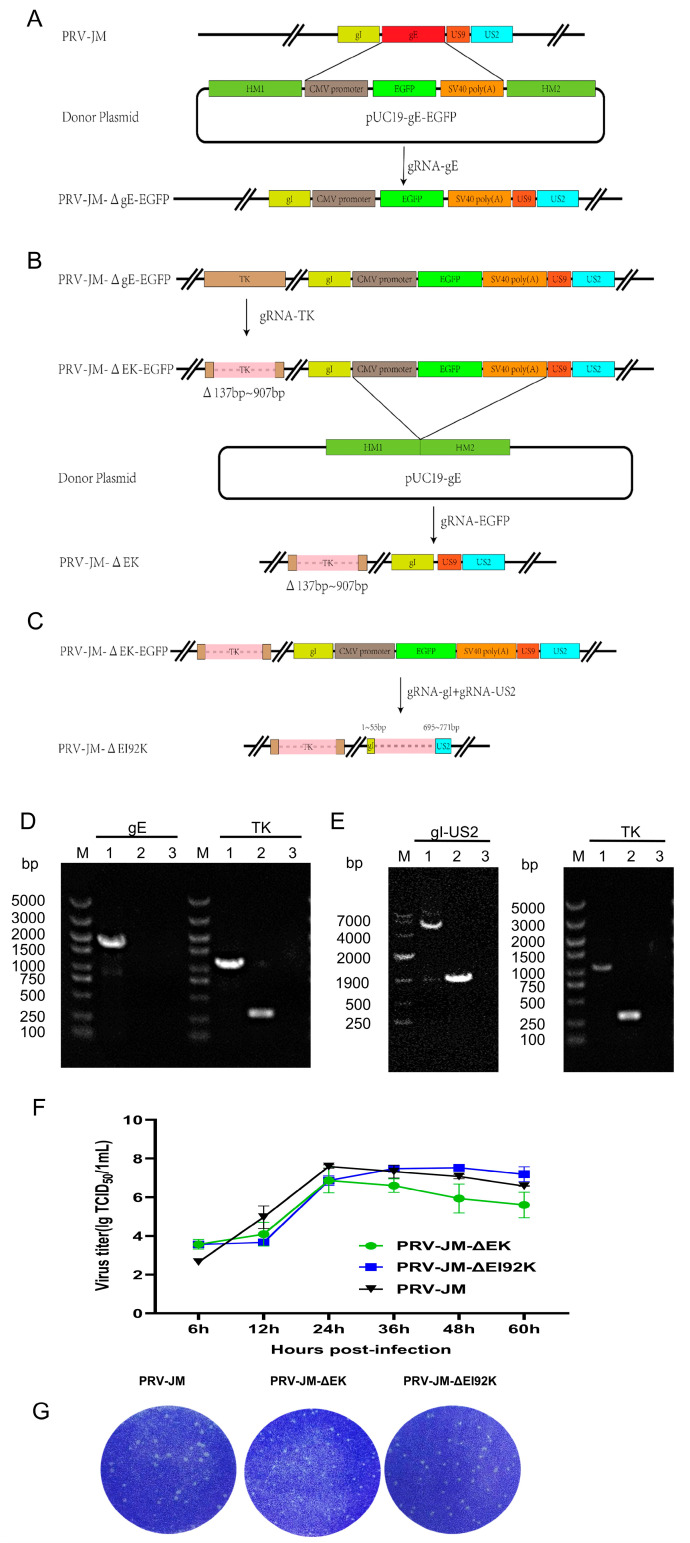
Construction strategy and growth characteristics of recombinant virus. (**A**) PRV-JM-Δ*gE*-EGFP, (**B**) PRV-JM-ΔEK, and (**C**) PRV-JM-ΔEI92K. After constructing PRV-JM-ΔEK and PRV-JM-ΔEI92K using homologous recombination method and CRISPR technology, PCR was performed using identification primers for identification according to strip size and showed that PRV-JM-ΔEK (**D**) and PRV-JM-ΔEI92K (**E**) were successfully constructed. (**A**) Line 1: PRV-JM genome; line 2: JM-ΔEK genome; line 3: blank control. (**B**) Line 1: PRV-JM genome; line 2: PRV-JM-ΔEI92K genome; line 3: blank control. (**F**) One-step growth curve of viruses. PK-15 cells were infected with PRV-JM, PRV-JM-ΔEK, or PRV-JM-ΔEI92K at MOI = 0.1. At designated time points, cells were harvested and viral titers determined on PK-15 cells. Results are representative of three independent experiments. (**G**) Plaque morphologies of PRV-JM, PRV-JM-ΔEK, or PRV-JM-ΔEI92K in PK-15 cells.

The strains were constructed according to the strategy in Figure 1. The PRV-JM genome, pUC19-*gE*-EGFP, and pX330-*gE* were co-transfected into Vero cells. The recombinant fluorescent virus PRV-JM-Δ*gE*-EGFP was purified and identified by PCR and sequenced (Figure S1A and Figure 1C). The PRV-JM-Δ*gE*-EGFP genome and pX330-*TK* were co-transfected into Vero cells, and then the recombinant virus PRV-JM-Δ*gE*/*TK*-EGFP was obtained (Figure S1B and Figure 1C). Finally, by co-transfecting the PRV-JM-Δ*gE*/*TK*-EGFP genome with pUC19-*gE*/pX330-EGFP and pX330-*gI*/pX330-*US2* into Vero cells, two recombinant viruses, PRV-JM-Δ*gE*/*TK* (PRV-JM-ΔEK) (Figure 1D) and PRV-JM-Δ*gE*/*gI*/*US2*/*US9*/*TK* (PRV-JM-ΔEI92K), were constructed (Figure 1E). Both recombinant viruses were consecutively cultured for 20 generations, and no gene complementation was found (Figure S1D,E).

### 3.2. Growth Characteristics of Recombinant Viruses

To assess the growth features of the two viruses, the one-step growth curves were analyzed, and the viral plaque-forming ability was examined through plaque assays. As shown in Figure 1F, PRV-JM-ΔEI92K and PRV-JM exhibited similar growth trends with comparable growth kinetics and viral titers, which was consistent with the conclusion reported previously [26]. However, the viral titer of PRV-JM-ΔEK was one titer lower than that of the wild-type strain PRV-JM. The plaque sizes of PRV-JM-ΔEI92K were smaller than those of PRV-JM-ΔEK, but both were smaller than those formed by the wild-type strain PRV-JM (Figure 1G). The lesions of infected cells showed that only PRV-JM-ΔEI92K did not form syncytia, while the cells infected with PRV-JM-ΔEK and PRV-JM formed syncytia of different sizes. These results indicated that the intercellular transmission of PRV-JM-ΔEI92K was lower than that of PRV-JM-ΔEK. Finally, both recombinant viruses were further propagated for 20 generations on PK-15 cells and then confirmed by PCR. As shown in Figure S1D–E, both recombinant viruses have good genetic stability.

### 3.3. Safety Experiment of Recombinant Virus in Mice

**Figure 2:**
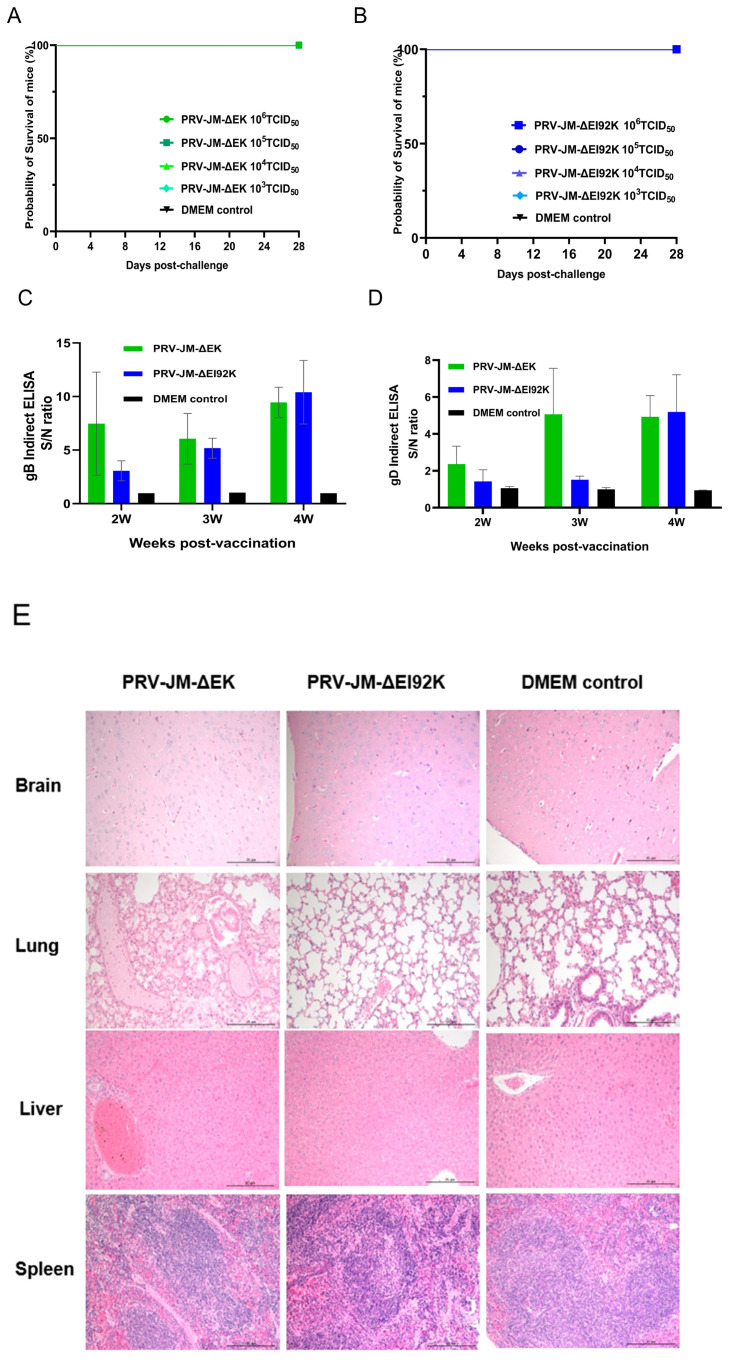
The safety of JM-ΔEK and JM-ΔEI92K in mice. (**A**,**B**) The survival curve of mice injected with different doses of PRV-JM-ΔEK and PRV-JM-ΔEI92K. The mice were randomly divided into 13 groups, with 5 mice in each group. And 8 groups were injected separately with different titers (10^3^TCID_50_, 10^4^TCID_50_, 10^5^TCID_50_, and 10^6^TCID_50_) of PRV-JM-ΔEK and PRV-JM-ΔEI92K via intramuscular injection. At the same time, a group of mice was injected with DMEM as a control. The situations of the survival and death of mice were monitored for 28 days after immunization. (**C**,**D**) The specific antibodies of gB and gD in mice at different time points after immunization. gB-specific antibodies and gD-specific antibodies were detected using an indirect ELISA method. The values for serum samples are given as signal/noise (S/N) ratios, and a higher S/N value means more antibodies. (**E**) The histological analysis of brains, livers, and lungs from mice injected with the indicated viral strains (10^6^TCID_50_ PRV-JM, 10^6^TCID_50_ PRV-JM-ΔEK, or 10^6^TCID_50_ PRV-JM-ΔEI92K) or DMEM. (Hematoxylin and eosin staining, 200× magnification).

The safety of the two recombinant viruses was evaluated in mice. Mice infected with PRV-JM (from 10^3^ to 10^6^ TCID_50_) developed severe pruritus symptoms, and all died within 4 days (Figure S2). In contrast, mice in the PRV-JM-ΔEK and PRV-JM-ΔEI92K groups (from 10^3^ to 10^6^ TCID_50_) did not show any clinical signs of illness and survived until the end of the observation period (Figure 2A,B). Additionally, we also noticed that the levels of gB- and gD-specific antibodies increased in all the vaccinated groups (Figure 2C,D), suggesting that PRV-JM-ΔEK or PRV-JM-ΔEI92K infection induced protective antibodies, which may provide effective protection against the virulent PRV challenge. The lesions of experimental mice in the 10^6^ TCID_50_ group were examined using pathological sections (Figure 2E). PRV-JM-ΔEK infection resulted in the accumulation of fibrinous exudate in the alveolar spaces, congestion in the hepatic veins and sinusoids, and mild hepatocellular degeneration. Simultaneously, no histopathological changes were observed in the brains, lungs, spleens, or livers of mice infected with PRV-JM-ΔEI92K or control mice injected with DMEM. These results showed that PRV-JM-ΔEI92K was safer than PRV-JM-ΔEK, even though neither was lethal to the mice.

### 3.4. Protective Experiment of Recombinant Virus in Mice

**Figure 3:**
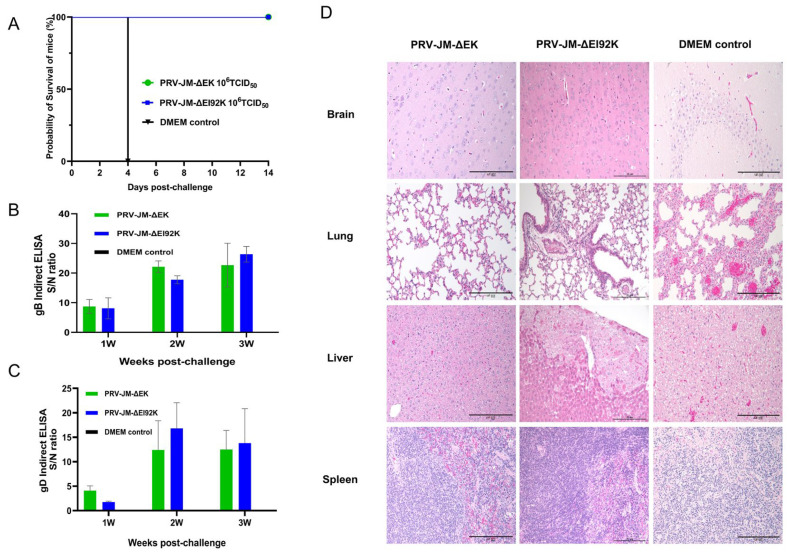
The protection of PRV-JM-ΔEK and PRV-JM-ΔEI92K in mice. (**A**) The survival curve of mice after challenging. The mice were randomly divided into 3 groups, with 5 mice in each group. They were injected intramuscularly with 10^6^ TCID_50_ of PRV-JM-ΔEK and PRV-JM-ΔEI92K and 100 μL DMEM. After 28 days of immunity, each mouse was injected intramuscularly with PRV-JM. (**B**,**C**) The specific antibodies of gB and gD in mice at different time points after the challenge. gB-specific antibodies and gD-specific antibodies were detected using an indirect ELISA method. The values for serum samples are given as signal/noise (S/N) ratios, and a higher S/N value means more antibodies. (**D**) The histological analysis of brains, livers, and lungs from the indicated groups (10^6^TCID_50_ PRV-JM-ΔEK, 10^6^TCID_50_ PRV-JM-ΔEI92K, or DMEM) after the challenge with PRV-JM. (Hematoxylin and eosin staining, 200× magnification).

To test the protection of the viruses, 10 mice were selected for vaccination with 10^6^ TCID_50_ of PRV-JM-ΔEK and PRV-JM-ΔEI92K. Five mice were injected with DMEM. Subsequently, mice were challenged with 10^4^ TCID_50_ of the PRV-JM strain at 28 dpi. All the control mice incubated with DMEM died at 4 dpc, but mice immunized with PRV-JM-ΔEK or PRV-JM-ΔEI92K survived during the observation period of 14 days (Figure 3A). These results indicate that the protection rate of PRV-JM-ΔEK and PRV-JM-ΔEI92K could reach 100% in mice. At the same time, we also found that the levels of gD- and gB-specific antibodies increased significantly after the challenge (Figure 3B,C), which indicated that PRV-JM-ΔEK or PRV-JM-ΔEI92K immunization could induce strong memory immunity. The surviving and dead mice were necropsied (*N* = 15). The control mice showed the degeneration of neurons in the hippocampus of the brain; the concentration of cell nuclei; the congestion of capillaries and venules in the alveolar walls; the mild thickening of the alveolar walls; extensive, diffuse, moderate-to-severe liver cell degeneration; and the blurred structure of splenic lymph nodes (Figure 3D). Simultaneously, lymphocyte levels decreased, as did hepatocyte degeneration in the PRV-JM-ΔEK group, but local perivascular edema in the lungs, mild inflammatory infiltration, and focal hepatocyte degeneration in the liver were seen in the PRV-JM-ΔEI92K group (Figure 3D). The results showed that PRV-JM-ΔEK and PRV-JM-ΔEI92K could provide effective protection for mice against the challenge of PRV.

### 3.5. Protection of Both Recombinant Viruses in Pigs

**Figure 4:**
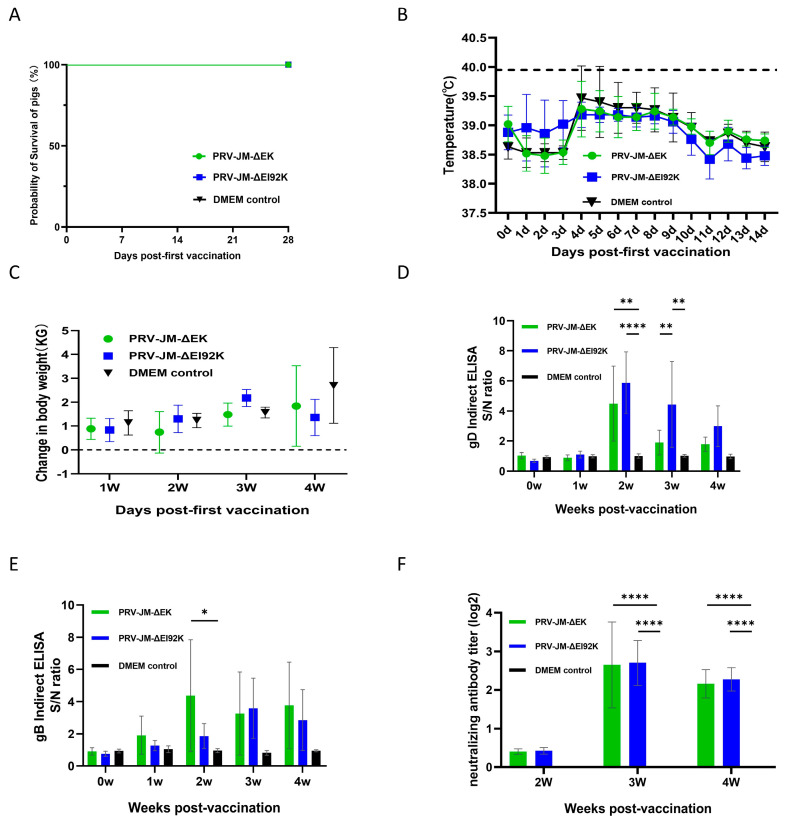
Pathogenicity of PRV-JM-ΔEK and PRV-JM-ΔEI92K in piglets. (**A**) Survival curves of piglets after challenge with indicated viral strains. (**B**) Daily rectal temperatures of all piglets after vaccination. (**C**) Weekly weight changes in all piglets after vaccination. (**D**) gD-specific antibodies and (**E**) gB-specific antibodies in different groups of piglets at indicated time points. PRV gB-specific antibodies and PRV gD-specific antibodies were detected using indirect ELISA method. Values for serum samples are given as signal/noise (S/N) ratios, and higher S/N value means more antibodies. (**F**) Serum neutralizing antibodies (NAbs) against PRV-JM strains in indicated groups at indicated time points. Standard deviations are shown as error bars (* *p* ≤ 0.05; ** *p* < 0.01; **** *p* < 0.0001).

To further investigate the protective immunity of recombinant viruses, pigs were injected intramuscularly with PRV-JM-ΔEK, PRV-JM-ΔEI92K, and DMEM culture medium. No clinical signs of illness related to pseudorabies were observed until challenge in pigs immunized with recombinant viruses, and no pigs died (Figure 4A,B). The average weight gain of pigs was not affected within four weeks after immunization (Figure 4C). Then, the serum of immunized pigs was detected using a *gE* commercial kit, which could distinguish the immunized pigs. The specific antibodies in the serum of the immune period against gD and gB were tested (Figure 4D,E). Interestingly, PRV-JM-ΔEK produced gB-specific antibodies earlier, whereas PRV-JM-ΔEI92K produced more intense gD-specific antibodies. It was found that the gD and gB antibodies peaked in the second to third weeks. The neutralizing antibodies of the pigs in the PRV-JM-ΔEK and PRV-JM-ΔEI92K vaccine groups, which were produced after 2 weeks post-vaccination and reached a maximum at 3 weeks post-vaccination, began to decay at 4 weeks post-vaccination (Figure 4F). As a negative control, pigs injected with DMEM did not produce any antibody or neutralizing antibody of PRV. These results showed that PRV-JM-ΔEK and PRV-JM-ΔEI92K as an attenuated vaccine can induce humoral immunity very early and reach a maximum at 3 weeks post-vaccination.

**Figure 5:**
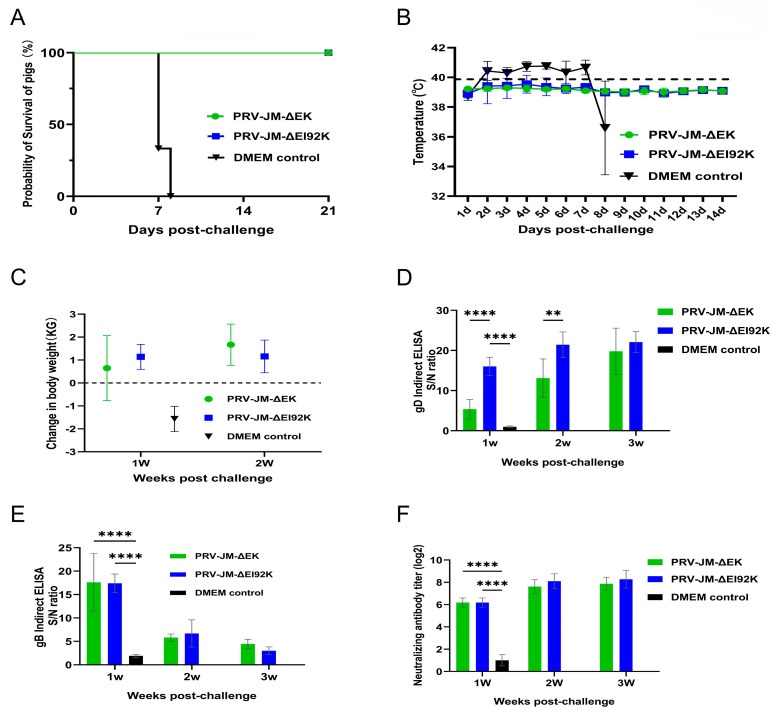

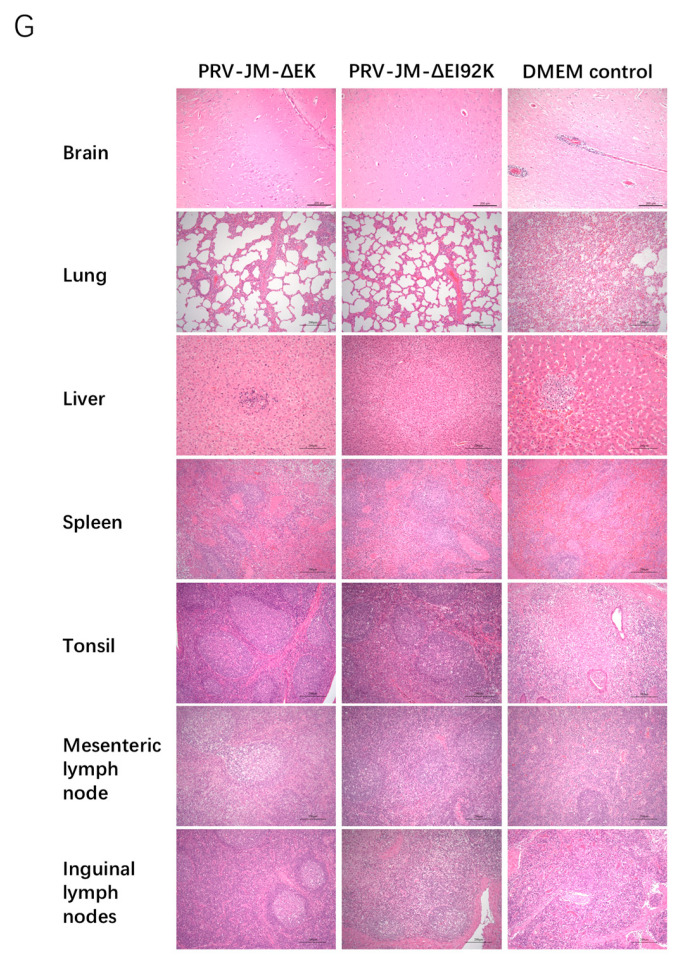
Protection of PRV-JM-ΔEK and PRV-JM-ΔEI92K in piglets. (**A**) Survival curves of pigs after challenge with PRV strain PRV-JM. (**B**) Rectal temperatures of piglets after challenge with PRV-JM. (**C**) Weekly weight changes in all piglets after challenge with PRV-JM. (**D**) gD-specific antibodies and (**E**) gB-specific antibodies in different groups of piglets at indicated time points. gB-specific antibodies and gD-specific antibodies were detected using indirect ELISA method. Values for serum samples are given as signal/noise (S/N) ratios, and higher S/N value means more antibodies. (**F**) Serum neutralizing antibodies (NAbs) against PRV-JM in indicated groups at indicated time points. Standard deviations are shown as error bars. (** *p* < 0.01; **** *p* < 0.0001) (**G**) Histological analysis of brains, livers, and lungs from pigs from indicated groups after challenge with PRV-JM. Arrows show viral encephalitis and neurophagocytosis in brain, inflammatory cell infiltration in liver, and inflammatory cell infiltration in alveolar walls of lungs (hematoxylin and eosin staining, 200× magnification).

After 28 days post-vaccination, pigs in each group were challenged with 2 × 10^5^ TCID_50_ of the wild-type PRV-JM strain through intranasal instillation, and the pigs were observed for 21 days. Compared with the pigs in the DMEM group, which died at 8 days post-challenge, no pigs in the vaccine group died during the observation period (Figure 5A). The protection rates of pigs vaccinated with PRV-JM-ΔEK and PRV-JM-ΔEI92K reached 100%. It was found that all the pigs did not have fever after the challenge, and their body temperature remained at about 39 °C (Figure 5B), without any clinical signs of illness, and they were in good health. Pigs kept gaining weight (Figure 5C). All the pigs in the DMEM group had a temperature rise of 41 °C on the second day after the challenge. After 6 days of high temperatures, their temperature dropped rapidly, and they died. During the high fever, clinical signs of illness such as itching, anorexia, drowsiness, and neurological symptoms of limbs appeared. The gD-specific antibodies rapidly increased over time after the challenge, but gB-specific antibodies were high in the first week after the challenge and decreased in the second week (Figure 5D,E). The PRV-JM-ΔEK- and PRV-JM-ΔEI92K-vaccinated groups exhibited similar levels of neutralization antibody titers against PRV-JM, and the titers of the control group were lower than 1:2 (Figure 5F).

A histopathological examination of pigs’ brains, livers, spleens, lungs, tonsils, and lymph nodes (*N* = 9) was performed 21 days after the challenge (Figure 5G). In the control group, there were obvious lesions. The lymph follicles of intestinal lymph nodes and abdominal lymph nodes atrophied, and architectural effacement and lymphoid depletion were observed. Lymphocytes in some lymphoid follicles in the tonsils were necrotic, the structure of lymphatic follicles was blurred and disappeared, and some alveolar spaces were atretic. Serous and fibrinous exudates could be seen in them, and the infiltration of inflammatory cells such as macrophages could also be seen. Inflammatory cell infiltration could be seen perivascularly in the cortex and white matter of the brain. There was focal inflammatory cell infiltration in the liver parenchyma, hepatocytes were replaced by inflammatory cells, and sinusoidal congestion occurred. The white pulp in the spleen atrophied, lymphocyte levels decreased, and there was congestion in the lungs. However, in the JM-ΔEK group, there was a slight decrease in lymphocytes in the lymphoid follicles of the intestinal lymph nodes and abdominal lymph nodes. In the brain, some perivascular inflammatory cuffing could be seen in the cortex, infiltrating and mildly thickening it. Focal hepatocellular degeneration and necrosis, inflammatory cell infiltration, and hypocellularity were seen in the spleen’s white pulp. No obvious changes were found in the jaw lymph nodes, tonsils, or lungs. In addition, the pathological sections of the JM-ΔEI92K group showed a decrease in abdominal lymphocytes, a small amount of glial cell infiltration in the cortex of the brain, mild hepatocellular degeneration, and a slight decrease in spleen white pulp lymphocytes. No obvious changes were found in jaw lymphadenitis, intestinal lymphadenitis, the tonsils, or lungs.

All these results indicate that both PRV-JM-ΔEK and PRV-JM-ΔEI92K can completely protect the pigs from the challenge of the wild-type PRV-JM strain. The pathological damage of the pigs in the PRV-JM-ΔEI92K group was milder, making it a good gene deletion vaccine and an ideal virus vector.

## 4. Discussion

Porcine pseudorabies (PR) is an acute and severe infectious disease that seriously harms the pig breeding industry. Pigs are the only natural host of the virus. Latent infection is not easily formed after infection in other animals. The main clinical signs of illness are itching, high fever, reproductive disorders, and encephalomyelitis [5,6]. PRV can also significantly cause immune suppression, cause vaccine immunity failure, and aggravate the mixed infection of bacteria and viruses [27,28]. Common viral mixed infections include porcine reproductive and respiratory syndrome virus (PRRSV), porcine circovirus type 2 (PCV2), etc. [10,11].

According to the number of virulence genes that are deleted, PRV attenuated vaccines are divided into single-gene, double-gene, and multi-gene deletion vaccines [29,30]. In 1985, the first single-gene deletion vaccine of PRV was developed using PRV strain BuK-d13 with a *TK* mutant [29]. Subsequently, Chen et al. successfully developed a PRV vaccine with *TK* gene deletion [30]. Yao et al. successfully developed a PRV vaccine with the deletion of the *gE* gene in 2011 [31]. Subsequently, Kit et al. developed a PRV live attenuated vaccine by constructing a PRV-Δ*TK*/*gE* double-gene deletion strain [32]. He et al. constructed a PRV-HB-98 strain with the deletion of the *TK* and *gC* genes [33]. Liang et al. developed a vaccine with the deletion of the *gE/TK* double gene [34]. And Tong et al. constructed PRV-*JS-2012*-△*gE*/*gI* vaccine candidate strains [35].

There are several methods for eliminating viral genes. However, the traditional bacterial artificial chromosome (BAC) method is complex and typically requires several months to construct the BAC system. Additionally, the recombinant Red ET system is employed to manipulate the BAC plasmid, but it is important to note that the vector fragment of BAC remains present in the final virus. Cas9 offers significant advantages, particularly in terms of rapid construction speed. While there may be challenges associated with the screening and identification of the virus, the absence of other gene fragments in the resulting virus enhances its reliability. Furthermore, by employing GFP as a marker, double-deletion and five-deletion strains can be generated through both positive and negative screening methods, allowing for the direct selection of viruses based on the presence or absence of fluorescence, thereby facilitating a more efficient identification of deleted viruses.

Currently, research indicates that PRV vaccines, which involve knocking out *gE* and *TK*, can effectively protect pigs from PRV infection. However, these vaccines may pose safety risks to other animals, including dogs and cats. *Bartha-K61* is a traditional vaccine virus characterized by the natural deletions of *gI*, *gE*, *US9*, and *US2*. This experiment combined the strategy of natural deletion with an additional deletion of *TK* to create a safe deleted strain. The live attenuated vaccine has good immune effects. However, attenuated vaccines often lack one or several key genes for virulence, and the strains may become virulent again during clinical use due to virus evolution and recombination. To reduce the probability of virulence reversal, the knockout strategy in this study involves deleting large fragments of genes rather than frameshift mutation. Consequently, the safety and protective efficacy of the two types of deleted viruses were compared in this study. Additionally, five-deficient poisoned pigs were found to be safe for rabbits. After the vaccine is immunized, the gene deletion vaccine can stimulate antibody production in pigs within the second week. Although the titer of neutralizing antibodies stimulated by deletion vaccine-immunized pigs is not high after immunization, the titer of neutralizing antibodies increases immediately after the challenge, and the antibodies can protect pigs effectively from PRV. Previous studies have shown that the deletion of *US2* can increase the titer of PRV in the primary culture system of the pig cerebral cortex [26]. By measuring the growth curve, this experiment found that the deletion of *US2* can still increase the titer in PK15. This is extremely beneficial to vaccine production.

The safety of live vaccines has always been a controversial topic, so it is very important to verify their safety. PRV can infect most mammals. Pigs are PRV’s natural hosts, and mice are more susceptible than pigs. Therefore, the mouse animal model is more suitable for studying the safety of PRV. The experimental results show that the safety of and difference in PRV-JM-ΔEK and PRV-JM-ΔEI92K are only seen in mouse slices, and there is no difference in mortality or clinical signs, indicating that the safety of the two viruses is not much different, but PRV-JM-ΔEI92K is safer than PRV-JM-ΔEK. Because mice are very sensitive to PRV, protective differences can also be seen in protective experiments, which are not obvious in pigs. This can also show the relationship between virulence and safety and protection, and there is a balance between virulence and safety and protection. High toxicity means worse safety but enhanced protection; low toxicity means better safety but lower protection. This balance is very important for vaccines, but it is very difficult to grasp the balance. This can make it easier for us to choose a better vaccine strain. Fortunately, PRV-JM-ΔEI92K has a higher virus titer but lower virulence than PRV-JM-ΔEK. PRV-JM-ΔEI92K is a promising candidate vaccine strain.

Although the results of this study are very good, there are still some limitations. First of all, the sheep sensitivity test is the most authoritative method for verifying the safety of PRV, which is irreplaceable. We will conduct this experiment later. Secondly, this experiment only carried out a virus attack experiment on the PRV-JM strain, and whether it has protection against other epidemic strains needs to be verified.

In summary, we used CRISPR/Cas9 technology to construct two PRV gene-deleted vaccine candidate strains, PRV-JM-ΔEK and PRV-JM-ΔEI92K. The two PRV gene-deleted vaccine candidate strains have efficacy, safety, and protection in mice and pigs. PRV-JM-ΔEI92K is the vaccine candidate strain with the most potential because of its excellent safety and high virus titer. At present, PRV-JM-ΔEI92K has only been preliminarily studied. Next, we will explore its cross-protection against the current domestic epidemic strains. It is hoped that PRV-JM-ΔEI92K will be a vaccine candidate for the prevention of the current epidemic of PR in China.

## 5. Conclusions

In summary, two PRV gene-deletion candidate vaccine strains with good safety and protection in mice and pigs were successfully constructed in this study, among which PRV-JM-ΔEI92K has better safety and a higher virus titer, and it is an excellent gene deletion vaccine candidate strain for mutant viruses. This provides sufficient material reserves for the prevention and control of PRV in China and also serves as a basis for developing effective polyvalent recombinant PRV-based carrier vaccines in the future.

## Data Availability

Data supporting the reported results are available in this article and in the Supplementary Materials.

## Supplementary Materials

The following supporting information can be downloaded at https://www.mdpi.com/article/10.3390/vaccines13040359/s1, Figure S1: Identification of PRV-JM-Δ*gE*-EGFP and PRV-JM-ΔEK-EGFP and stability of PRV-JM-ΔEK and PRV-JM-ΔEI92K; Figure S2: The survival curve of mice injected with PRV-JM; Table S1: sgRNAs used in this study; Table S2: Oligonucleotide primers used for constructing homology arms in this study; Table S3: Oligonucleotide primers used for identifying recombinant viruses in this study.
