# Supplementary material for: Construction of Two Recombinant Pseudorabies Viruses with Deletion of Virulence Genes and Evaluation of Their Immune Protection in Mice and Piglets
Source: Vaccines (Basel). 2025 Mar 27;13(4):359. doi: 10.3390/vaccines13040359 (PMC12030885; doi:10.3390/vaccines13040359)
Supplement: Supplementary file 1 [file vaccines-13-00359-s001.zip › vaccines-3534955-supplementary.pdf]

Supplementary data

Construction of two recombinant pseudorabies viruses with deletion of virulence genes and evaluation of their immune protection in mice and piglets

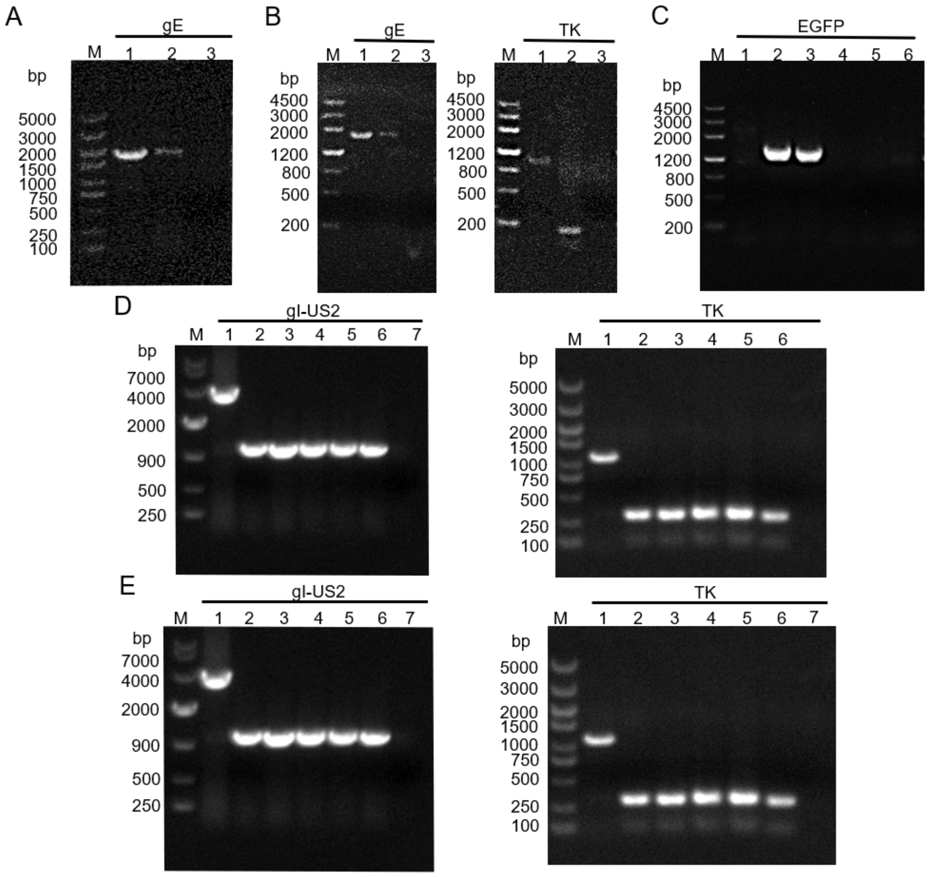

Figure S1. Identification of PRV-JM-ΔgE-EGFP and PRV-JM-ΔEK-EGFP and stability of PRV-JM-ΔEK and PRV-JM-ΔEI92K

(A) The construction of JM-ΔgE-EGFP was successfully identified by PCR using gE primers. (line 1:PRV-JM genome; line 2: PRV-JM-ΔgE-EGFP genome; line 3: blank control) (B) The construction of PRV-JM-ΔEK-EGFP was successfully identified by PCR using gE and TK primers. (line 1:PRV-JM genome; line 2: PRV-JM-ΔEK-EGFP genome; line 3: blank control) (C) The strains containing PRV-JM-ΔgE-EGFP, PRV-JM-ΔEK-EGFP, PRV-JM-ΔEK, and PRV-JM-ΔEI92K were further identified by PCR with EGFP identification primer. (line 1:PRV-JM genome; line 2: PRV-JM-ΔgE-EGFP genome; line 3: PRV-JM-ΔEK-EGFP genome; line 4: PRV-JM-ΔEK genome; line 5: PRV-JM-ΔEI92K genome; line 6: blank control)（D,E）Identification and genetic stability of the recombinant PRV-JM-ΔEK and PRV-JM-ΔEI92K assessed by specific PCR. (line 1: PRV-JM genome; line 2: P1 line 3: P5; line 4: P10; line 5: P15; line 6: P20; line 7: blank control)

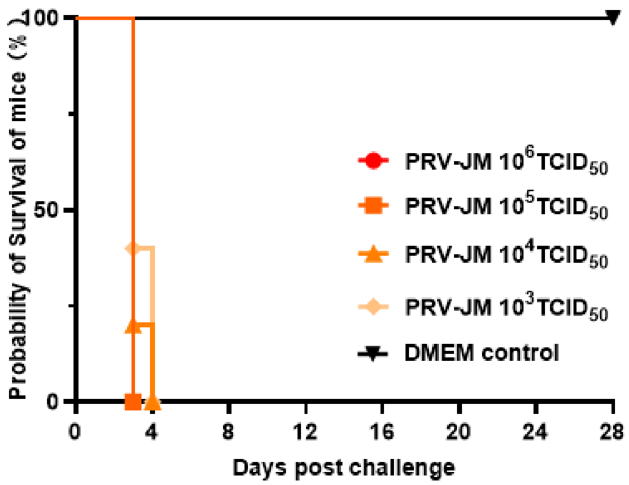

Figure S2. The survival curve of mice injected with PRV-JM.

The survival curve of mice injected with different doses of PRV-JM (10^3TCID$_{50}$, 10^4TCID$_{50}$, 10^5TCID$_{50}$, and 10^6TCID$_{50}$). At the same time, a group of mice was injected with DMEM as a control.

Table S1. sgRNAs used in this study.

sgRNA Primers	Primer sequences (5’-3’)
gE sgRNA-F	CACCGGGCAGGAACGTCCAGATCC
gE sgRNA-R	AAACGGATCTGGACGTTCCTGCCC
TK sgRNA1-F	CACCTCTGTTCGACACGGACACGG
TK sgRNA1-R	AAACCCGTGTCCGTGTCGAACAGA
TK sgRNA2-F	CACCCCGGATGTGGTCGCCGTACG
TK sgRNA2-R	AAACCGTACGGCGACCACATCCGG
US2 sgRNA-F	CACCCACTCCCAGATCGTGACCCG
US2 sgRNA-F	AAACCGGGTCACGATCTGGGAGTG
gI sgRNA-F	CACCCAGGGCGGCCAGGGTCAGGG
gI sgRNA-R	AAACCCCTGACCCTGGCCGCCCTG
EGFP sgRNA-F	CACCGGGCGAGGAGCTGTTCACCG
EGFP sgRNA-R	AAACCGGTGAACAGCTCCTCGCCC

Table S2. Oligonucleotide primers used for constructing homology arms in this study.

Primers	Primer sequences (5’-3’)
gE-HM1-F	GCTATGACCATGATTACGCCAAGCTTGCAGCCCGGTCCGTAGC
gE-HM1-R	GCTATGACCATGATTACGCCAAGCTTGCAGCCCGGTCCGTAGC
gE-HM2-F	GTCCAAACTCATCAATGTATCTTAATACCGGGAGAACCGGTC
gE-HM2-R	GAGCTCGGTACCCGGGGATCCGAGAGATCCTGCCGTCTAGGA

Table S3. Oligonucleotide primers used for identifying recombinant viruses in this study.

Primers	Primer sequences (5’-3’)
gE-F	CGCGAGTCTCGCACACAC
gE-R	TCGGAATGCGGGCGGAC
TK-F	ATGCGCATCCTCCGGATCT
TK-R	CATACACATGGCTTTATACGCG
EGFP-F	CGTTACATAACTTACGGTAAATGGCCC
EGFP-R	TAAGATACATTGATGAGTTTGGACAAAC
